# Supplementary material for: Stakeholder perspectives on the barriers and facilitators to integrating cardiovascular disease and diabetes management at primary care in Kenya
Source: PLOS Glob Public Health. 2025 Jul 3;5(7):e0004164. doi: 10.1371/journal.pgph.0004164 (PMC12225785; doi:10.1371/journal.pgph.0004164)
Supplement: S1 Text — (DOCX) [file pgph.0004164.s001.docx]

**S1 Text: Key informant interview guide**

**[Interviewer] In this interview, we will discuss questions related to national health priorities for cardiovascular diseases, diabetes and multimorbidity, and the application of health system interventions including integrated care and multicomponent interventions to control these conditions.**

1. Please describe your current role and what it entails?
2. To start, could you briefly give a picture of the policy landscape for cardiovascular diseases and diabetes in Kenya?
3. What is the process of priority setting for non-communicable diseases in Kenya?
   1. [Probe] Planning and budgeting for NCDs, cardiovascular diseases and diabetes (What influences budget allocation for CVD prevention? - Barriers and facilitators, recommendations)
   2. In your opinion, is cardiovascular diseases and diabetes prevention and management prioritised in the national health policy and strategic plan in Kenya?
      1. What aspects are prioritised?
4. What is the place of comorbidity and/ or multimorbidity of these conditions (an increasingly important health issue in LMICs)?
5. In your opinion, what specific interventions or strategies should be / are being prioritised to improve the prevention and management of cardiovascular diseases and diabetes in Kenya?
   1. [Probe] Interventions targeting single diseases, comorbidity and/ or multimorbidity?
   2. [Probe] What levels of the health system are they implemented? (Primary, Secondary… etc.)
6. Integrated care is one of the recommended interventions in prevention, management, and control of chronic diseases. In the Kenyan context, what does integrated care mean?
7. In your view, do you think using integrated care can help in the prevention, control and management of cardiovascular diseases and diabetes or their co/multimorbidity in the Kenyan context? **[Follow-up probe]** Why?
   1. [Probe] Are you aware of any integrated care interventions that are currently being implemented by the government for cardiovascular disease/ diabetes/ their co/multimorbidity in Kenya? **[Follow-up probe]** What are they?
   2. [Probe] What do you think are the barriers to the implementation of integrated care interventions for cardiovascular diseases/ diabetes/ co/multimorbidity in Kenya?
   3. [Probe] What factors do you think could promote effective implementation of integrated care interventions for cardiovascular diseases/ diabetes/ co/multimorbidity in Kenya?
   4. [Probe] What considerations should be made when scaling up integrated care for cardiovascular diseases/ diabetes/ co/multimorbidity in Kenya?
8. Do you have any final comments on what we have discussed today?

**Thank you for participating in this interview**
