# Supplementary material for: Stakeholder perspectives on the barriers and facilitators to integrating cardiovascular disease and diabetes management at primary care in Kenya
Source: PLOS Glob Public Health. 2025 Jul 3;5(7):e0004164. doi: 10.1371/journal.pgph.0004164 (PMC12225785; doi:10.1371/journal.pgph.0004164)
Supplement: S1 Table — (DOCX) [file pgph.0004164.s002.docx]

**S1 Table: Definition of the rainbow model of integrated care framework elements**

| **Dimensions** | **Definition of key elements** |
| --- | --- |
| **Integrated care** |  |
| Horizontal integration: | Relates to strategies that link similar levels of care |
| Vertical integration: | Relates to strategies that link different levels of care |
| System integration | Refers to the alignment of rules and policies within a system |
| Organisational integration: | Refers to the extent to which organisations coordinate services across different organisations |
| Professional integration: | Refers to extent to which professionals coordinate services across various disciplines |
| Clinical integration: | Refers to the extent to which care services are coordinated |
| Functional integration: | Refers to the extent to which back-office and support functions are coordinate |
| Normative integration: | Refers to the extent to which mission, work values etc. are shared within a system |
| **Primary care** |  |
| First contact care: | Implies accessibility to and use of services for each new problem or new episode of a problem for which people seek health care |
| Continuous care: | Longitudinal use of a regular source of care over time, regardless of the presence or absence of disease or injury |
| Comprehensive care: | The availability of a wide range of services in and their appropriate provision across the entire spectrum of types of needs for all but the most uncommon problems in the  population. |
| Coordinated care: | The linking of health care events and services so that the patient receives appropriate care for all his/her health problems, physical as well as mental and social. |
