# Supplementary material for: Stakeholder perspectives on the barriers and facilitators to integrating cardiovascular disease and diabetes management at primary care in Kenya
Source: PLOS Glob Public Health. 2025 Jul 3;5(7):e0004164. doi: 10.1371/journal.pgph.0004164 (PMC12225785; doi:10.1371/journal.pgph.0004164)
Supplement: S2 Table — (DOCX) [file pgph.0004164.s003.docx]

**S2 Table: Codebook for thematic analysis**

| **Health systems level** | **Dimensions of the Rainbow model of integrated care (RMIC)** | **Emerging themes** | |
| --- | --- | --- | --- |
|  |  | **Barriers** | **Facilitators** |
| **Macro level** | **System integration** |  |  |
| **Meso level** | **Organisational Integration** |  |  |
|  | **Professional integration** |  |  |
| **Micro level** | **Clinical integration** |  |  |
| **Linking Micro/ meso/ macro levels** | **Normative integration** |  |  |
|  | **Functional integration** |  |  |
